# Epidemiology of allergic rhinitis in Korean children

**DOI:** 10.1186/1939-4551-8-S1-A41

**Published:** 2015-04-08

**Authors:** Dae Hyun Lim

**Affiliations:** 1 Inha University Hospital, South Korea

## Background

Allergic rhinitis (AR) is a most common disease among allergic diseases in Korean children. We designed to investigate the rate and kinds of sensitized allergen in children with allergic rhinitis.

## Methods

The subjects were 12,469 school students (6,440 girls and 6,029 boys) in 5 areas (Incheon city, Busan city, Gwangju city, Kyoungki-do, Choongchungbook-do) from 2011 to 2014. Allergic rhinitis have diagnosed with positive ‘current symptoms and sign for allergic rhinitis’ on Korean ISAAC questionnaire and 1 more than proven allergen by skin prick test. Elementary school students were tested for 20 common aeroallergens, whereas middle and high school students were tested for 25 allergens. The 27 allergens included *Dermatophagoides pteronyssinus*, *Dermatophagoides farinae*, pollen (birch, alder, oak, Japanese cedar, pine, willow, elm, maple, bermuda grass, timothy grass, rye grass, orchard grass, meadow grass, vernal grass, mugwort, Japanese hop, fat hen, ragweed, and plantain), pets(dog and cat), and mold (*Penicillium*, *Aspergillus*, *Cladosporium*, and *Alternaria*).

## Results

The prevalence of AR is 21.9% (18.0% in girl and 25.5% in boy) in enrolled children, 12.6% (10.7% in girl and 14.5% in boy) in kindergarten, 21.0% (16.7% in girl and 25.0% in boy) in elementary school, 23.8% (18.5% in girl and 28.2% in boy) in middle school, 24.1 % (21.4% in girl and 26.8% in boy) in high school. Sensitization rate of allergen in students with AR was *D.pteronyssinus* (77.0%), *D.farinae* (68.2%), pollen (37.6%), molds (12.4%). Sensitization rate of cat and dog are 12.5% and 3.2%.

## Conclusions

This is the first data of prevalence of AR in Korean children. AR in Korean boy is more prevalent than in girl. Perennial AR is more frequent than seasonal type.

